# Identifying the Risk of Sepsis in Patients With Cancer Using Digital Health Care Records: Machine Learning–Based Approach

**DOI:** 10.2196/37689

**Published:** 2022-06-15

**Authors:** Donghun Yang, Jimin Kim, Junsang Yoo, Won Chul Cha, Hyojung Paik

**Affiliations:** 1 AI Technology Research Center Division of S&T Digital Convergence Korea Institute of Science and Technology Information Daejeon Republic of Korea; 2 Department of Data and High Performance Computing Science University of Science and Technology Daejeon Republic of Korea; 3 Center for Supercomputing Applications Division of National Supercomputing Korea Institute of Science and Technology Information Daejeon Republic of Korea; 4 Department of Digital Health Samsung Advanced Institute for Health Science & Technology Sungkyunkwan University Seoul Republic of Korea; 5 Department of Emergency Medicine Samsung Medical Center Sungkyunkwan University School of Medicine Seoul Republic of Korea

**Keywords:** sepsis, cancer, EHR, machine learning, deep learning, mortality rate, learning model, electronic health record, network based analysis, sepsis risk, risk model, prediction model

## Abstract

**Background:**

Sepsis is diagnosed in millions of people every year, resulting in a high mortality rate. Although patients with sepsis present multimorbid conditions, including cancer, sepsis predictions have mainly focused on patients with severe injuries.

**Objective:**

In this paper, we present a machine learning–based approach to identify the risk of sepsis in patients with cancer using electronic health records (EHRs).

**Methods:**

We utilized deidentified anonymized EHRs of 8580 patients with cancer from the Samsung Medical Center in Korea in a longitudinal manner between 2014 and 2019. To build a prediction model based on physical status that would differ between sepsis and nonsepsis patients, we analyzed 2462 laboratory test results and 2266 medication prescriptions using graph network and statistical analyses. The medication relationships and lab test results from each analysis were used as additional learning features to train our predictive model.

**Results:**

Patients with sepsis showed differential medication trajectories and physical status. For example, in the network-based analysis, narcotic analgesics were prescribed more often in the sepsis group, along with other drugs. Likewise, 35 types of lab tests, including albumin, globulin, and prothrombin time, showed significantly different distributions between sepsis and nonsepsis patients *(*P*<*.001). Our model outperformed the model trained using only common EHRs, showing an improved accuracy, area under the receiver operating characteristic (AUROC), and F1 score by 11.9%, 11.3%, and 13.6%, respectively. For the random forest–based model, the accuracy, AUROC, and F1 score were 0.692, 0.753, and 0.602, respectively.

**Conclusions:**

We showed that lab tests and medication relationships can be used as efficient features for predicting sepsis in patients with cancer. Consequently, identifying the risk of sepsis in patients with cancer using EHRs and machine learning is feasible.

## Introduction

Sepsis is a life-threatening organ dysfunction in which a pathogen infection leads to a dysregulated host response to the infection [1]. Sepsis is diagnosed in millions of people every year globally, accounting for a high ratio of in-hospital mortality (25%-50%) [2]. In particular, the mortality rate increases dramatically when septic shock is established [3,4]. Although a timely diagnosis of sepsis is essential for a promising prognosis, only minor cold-like symptoms, such as fever, excessive breathing, and increased pulse rate, are presented in the early stage of sepsis [5]. Therefore, in hospitals, patients admitted to the ward may suffer from septic shock after clinicians have missed the signature symptoms of sepsis. Thus, it is important to stratify high-risk patients and provide appropriate treatment in a short amount of time [6].

Sepsis has shown a substantial incidence in patients with low immunity, such as patients with cancer, patients who are elderly, and newborns [7]. Patients with cancer are at high risk for sepsis, as many are immunosuppressed due to the cancer itself and chemotherapy treatment [8]. For example, leukocyte counts are lowered, especially when anticancer treatments decrease bone marrow function, suppressing immune response to the pathogen [9]. Although predicting sepsis in patients with cancer is essential, an early identification of the risk of sepsis remains an unmet medical need.

Various studies have been conducted to identify the risk of sepsis, including a statistical model–based approach for emergency room (ER) patients [10], a machine learning–based approach for inpatients [11], and an approach using unstructured clinical data [12]. The majority of previous studies have focused on patients with severe trauma in the intensive care unit (ICU). However, the stratification of sepsis risk among patients with cancer has scarcely been conducted.

Our study aimed to predict the risk of sepsis in patients with cancer at an early stage using clinical information and a machine learning approach. We utilized the deidentified electronic health records (EHRs) from the Samsung Medical Center (SMC) in Korea of 8580 patients with cancer, including inpatients, outpatients, ICU patients, and ER patients. Drug prescriptions and laboratory test results are known to reflect the physical status of patients [13]. In our previous study, we showed that distributions of lab test results recapitulate the physical states of patients, including disease signatures and drug-associated responses [14]. Prescriptions of medications for cancer are mainly determined based on the patients’ medical conditions. Thus, we hypothesized that the patterns of prescribed medications and lab test results would be different between the sepsis and nonsepsis groups. To validate our hypothesis, we analyzed 2462 lab test results and 2266 medication prescriptions using network-based association rule [15] analysis and statistical analysis.

Based on the results of the analyses, we propose a machine learning–based sepsis predictive model that can reflect the physical conditions of patients with cancer and is trained on the prescribed drug and lab test patterns as well as EHRs, which are widely used in the reported sepsis prediction approaches [16,17].

## Methods

### Study Sample

Data were prepared from the Clinical Data Warehouse (CDW) and the SMC cancer registry, Seoul, South Korea, and deidentification was performed on the collected data. The study population included adult patients diagnosed with lung, liver, and breast cancer who visited the ER within 5 years of being diagnosed with cancer. The inclusion criteria were patients with cancer registered at the study sites. Patients were excluded from the study cohort if they met the following exclusion criteria: those under 18 years of age, those with multiple cancers, those who had not visited the emergency room within 5 years after the first cancer diagnosis, and those with ICD-10 codes not matched with C22, C34, and C50. The data were constructed by reflecting various EHR information such as hospitalization data, diagnosis code of cancer or other underlying disease, vital signs, genomic information, medication prescription, surgical history, radiation treatment, and lab test information for 5 years (2014-2019) before and after the cancer diagnosis of 8580 patients with cancer, including inpatients, outpatients, ICU patients, and ER patients. Most of the currently published sepsis prediction models use information within 48 hours before the onset of sepsis. However, due to the high risk of sepsis, it was considered necessary to predict in advance, so information 2 days prior to the ER visit was used. Data earlier than 7 days were somewhat difficult to consider as having an effect on the onset of sepsis, so the filtration criteria was set to 2-7 days.

### Ethics Approval

The institutional ethics committee of SMC approved this study (Institutional Review Board File #2019-06-071).

### Identifying Patients With Sepsis

We identified patients with sepsis using the Sequential Organ Failure Assessment (SOFA) scores of Third International Consensus Definitions for Sepsis and Septic Shock (Sepsis-3) guidelines [18] for a total of 18,610 ER visits by 8580 patients with cancer using the following procedures:

1. Nursing records, inspection records, clinical information, and medication prescription data were extracted from the CDW.
2. The variables were preprocessed to obtain the SOFA scores.
3. SOFA scores for each patient were calculated each time.
4. The time window was set by checking whether antibiotics were administered intravenously within 24 hours before and after the bacterial culture test.
5. Patients with sepsis were identified if their SOFA score changed by 2 points or more within the time window.
6. In accordance with the Sepsis-3 guidelines, if the SOFA score could not be measured in advance, it was considered 0. Consequently, if the change in the SOFA score was 2 or higher in the first visit to the ER, the patient was considered to be experiencing sepsis.

### Data Filtering and Preprocessing

We aligned the collected EHRs of 8580 patients with cancer based on the date of the ER visit and filtered patients with information 2-7 days prior to the ER visit. Each patient's diagnostic code was recorded as an ICD-9 code and standardized to 3 digits for use as a categorical feature. Because there was a possibility of information leakage from giving hints to the machine learning–based predictive model, lab test results centered on specific disease groups were removed, and only the lab test information performed on over 60% of the patients was used. All categorical features were preprocessed using one-hot encoding, and all binary categorical features were encoded as 0 or 1. In addition, missing values were imputed with the mean value of patients with the same type of cancer, the same sex, and the same age, and extreme outlier values were removed.

### Graph Network–Based Association Rule Analysis

Graph network–based association rules were performed on 2266 drug prescriptions. An association rule is a method for discovering frequent patterns and relationships between items from complicated data and can be employed to conceptualize complex dynamic systems comprising each interacting event [15]. Using the frequent pattern growth (FP-growth) algorithm [19], frequent relationships of drugs prescribed on the same day were analyzed. Next, only the group sets with a minimum support value of 0.05 or greater were selected. The support value (*S(D_i →_ D_j_*)), defined as in Equation (1), implied how often the sets go together when items are being tied up simultaneously, where *N(s)* represents the total number of prescriptions, and *N (D_i_,D_j_)* represents the number of events in which the *i-th* and *j-th* drugs were prescribed on the same day.



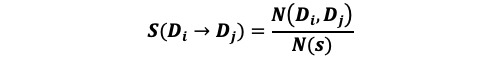



Finally, after designating each selected drug as a node, we plotted a graph network to visualize the result of the association rule analysis. The edges depicted the correlations of each drug.

### Vectorization of Prescribed Medication Relationships

We vectorized the relationships found through graph network–based association rule analysis to be used as an input for the machine learning–based sepsis prediction model. After multiplying each one-hot encoded drug selected through the aforementioned analysis by the number of prescription days, the relationship for each pair of values was vectorized using the 3 formulas proposed in our previous study [20]. These 3 formulas (*r*(*I, H, T*)) comprised the interaction (*I*), the harmonized average (*H*), and the arctangent (*T*), in which (*I*) determined the level of interaction, (*H*) determined the overall intensity in a sensitive manner, and (*T*) determined the geometric angle difference as a single scalar value for each pair, defined as in Equation (2), where D*_i_*^(^*^p,s)^* and D*_j_*^(^*^p,s)^* indicate the *i-th* and *j-th* drug of the *s-th* prescriptions for the *p-th* patient, respectively. The *D* value represents the prescription frequency of each medication.



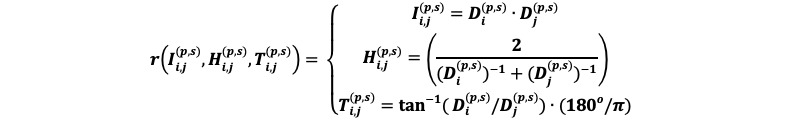



### Prediction of Sepsis Using Machine Learning Approaches

We trained models on vectorized drug relationships and selected lab test types, along with the common EHRs that are widely used in the reported sepsis prediction models [16,17]. We considered 2 machine learning models comprising logistic regression (LR) [21] and random forest [22] and 3 deep learning models comprising artificial neural networks (ANNs) [23], residual convolutional neural networks (ResNet10) [24], and long short-term memory recurrent neural networks (RNN-LSTMs) [25]. When applied to the model, the data were reshaped to (1, 42, 42) for the ResNet10 model and padded to the maximum length of the sequence and reshaped to (number of patients, time sequence, number of features) for the LSTM model. We investigated the important features using Shapley Additive Explanations (SHAP) [26]. SHAP, one of the Explainable Artificial Intelligence (XAI) techniques, is a method used to interpret results from deep learning and machine learning models and is based on game theory. We used Tree SHAP explainer to calculate the Shapley values.

All proposed approaches were implemented using the Python 3.7 library, such as PyTorch 1.5, Scikit-learn, and SHAP, on an NVIDIA TITAN RTX 24 GB × 2. The source code is available on GitHub [27].

## Results

### Characteristics of the Filtered and Preprocessed Data Set From SMC

**Figure 1:**
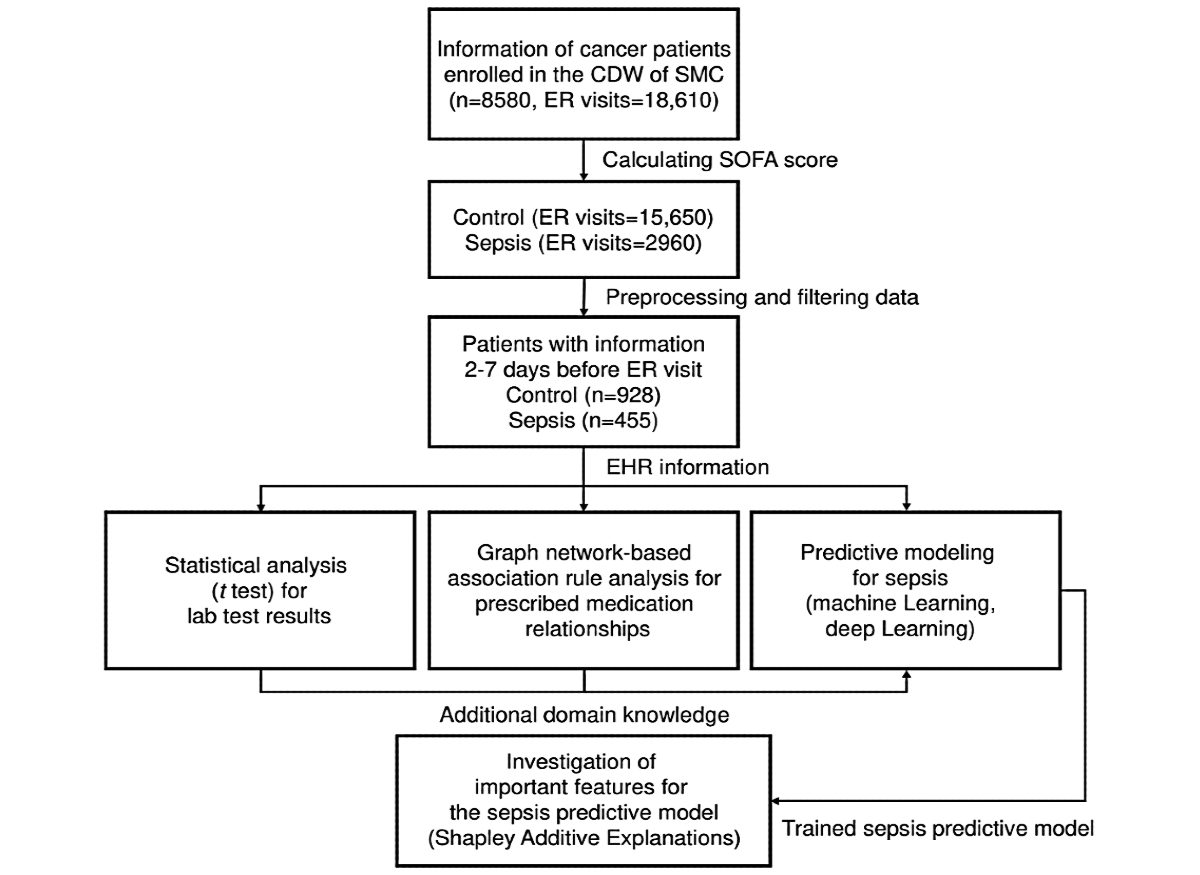
Study overview. CDW: Clinical Data Warehouse; EHR: electronic health record; ER: emergency room; ER visits: total number of ER visits by the patients; SOFA: Sequential Organ Failure Assessment of the Sepsis-3 guidelines; SMC: Samsung Medical Center.

The overall process of our study is shown in Figure 1. We analyzed data from 8580 patients obtained from the CDW of SMC. Using the SOFA scores of the Sepsis-3 guidelines, of a total of 18,610 ER visits by 8580 patients with cancer, 2960 visits were identified as sepsis and 15,650 visits as nonsepsis. As a result of filtering the patients, the control group included 928 patients, and the sepsis group included 455 patients. The statistics of the filtered and preprocessed data set that was used to build the sepsis predictive model are shown in Table 1.

In the control group (ie, nonsepsis patients with cancer), there were 490 (52.8%) males and 438 (47.2%) females. The mean age was 58.2 (SD 11.0) years, and the average weight was 63.7 (SD 10.7) kg. In terms of the initial cancer diagnosis of each patient, 180 (19.4%) had liver cancer, 533 (57.4%) had lung cancer, and 215 (23.2%) had breast cancer. Meanwhile, in the sepsis group, there were 324 (71.2%) males and 131 (28.8%) females, with a relatively higher proportion of males than the control group. The mean age of the sepsis group was 60.3 (SD 0.5) years, and the average weight was 64.3 (SD 11.3) kg. In the sepsis group, 140 (30.8%) patients had liver cancer, 274 (60.2%) had lung cancer, and 41 (9%) had breast cancer. With these prepared data sets from SMC, we analyzed the differences in medication patterns by group.

**Table 1:** Statistics of the input data used to build the sepsis predictive model.

Patient characteristics		Total (N=1383)	Control group (n=928)	Sepsis group (n=455)
**Sex, n (%)**				
	Male	814 (58.9)	490 (52.8)	324 (71.2)
	Female	569 (41.1)	438 (47.2)	131 (28.8)
	Age (years), mean (SD)	58.9 (10.9)	58.2 (11)	60.3 (0.5)
	Weight (kg), mean (SD)	63.9 (0.9)	63.7 (10.7)	64.3 (11.3)
**Cancer, n (%)**				
	Liver	320 (23.1)	180 (19.4)	140 (30.8)
	Lung	807 (58.4)	533 (57.4)	274 (60.2)
	Breast	256 (18.5)	215 (23.2)	41 (9.0)
Emergency room visits, n (%)		1466 (100)	991 (68)	475 (32)

### Graph Network–Based Association Analysis for Prescribed Medications

**Figure 2:**
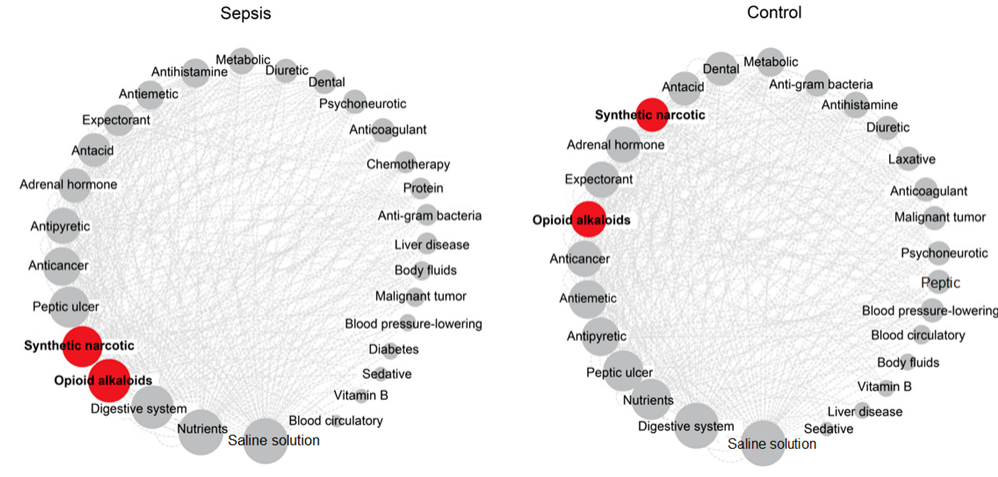
Results of the graph network-based association analysis for prescribed medications. Graph networks for sepsis (n=29) and control (n=28) patients. Each node represents the medication selected through association rule analysis (support value ≥ 0.05), and edges depict that the linked nodes were prescribed together. Red nodes and bars represent the drugs with different patterns between the sepsis and control patients.

Using the FP-growth algorithm, we analyzed patterns of the medications prescribed on the same day in 2666 prescriptions from the preprocessed and filtered EHR data. According to the analysis results, only group sets with a minimum support value of 0.05 or greater were selected. Of a total of 101 different drug types, 406 relationships among 29 drugs and 378 relationships among 28 drugs were selected for the sepsis group and nonsepsis group, respectively. To visualize the associations between the drug prescriptions, we constructed 2 graph networks with nodes representing the selected drugs and edges depicting the relationships among the nodes (Figure 2). The size of a node was determined by its average shortest path distance (Multimedia Appendix 1, graph A) and the number of edges (Multimedia Appendix 1, graph B), representing the topological properties of the network. A larger node meant that the corresponding drug was prescribed more often with other drugs compared to small nodes.

The patterns of medications between the sepsis and control groups were different. Nodes for medications such as “saline solution,” which are commonly prescribed for most patients, showed similar patterns in both networks (Figure 2), whereas “opioid alkaloids” and “synthetic narcotic” nodes were ranked higher in the sepsis group than in the control group (Multimedia Appendix 1, graphs A and B). These kinds of narcotic analgesic nodes were the bottleneck and central nodes, meaning that they were prescribed more often with other drugs in the sepsis group. Thus, we were able to confirm our hypothesis that relationships and patterns of prescribed medications were distinct in the 2 groups.

### Statistical Analysis for Lab Tests

Using a *t* test, we analyzed the 2462 lab test results of the preprocessed and filtered EHR data to find lab test items with significantly different distributions between the sepsis and nonsepsis groups. Multimedia Appendix 2 presents the means and standard deviations of the 2 groups for all lab test types, where the *P* is symbolized (no significance: NS, *P*<.05: *, *P*<.001: ***, *P*<.005: **, *P*<.001: ****).

**Figure 3:**
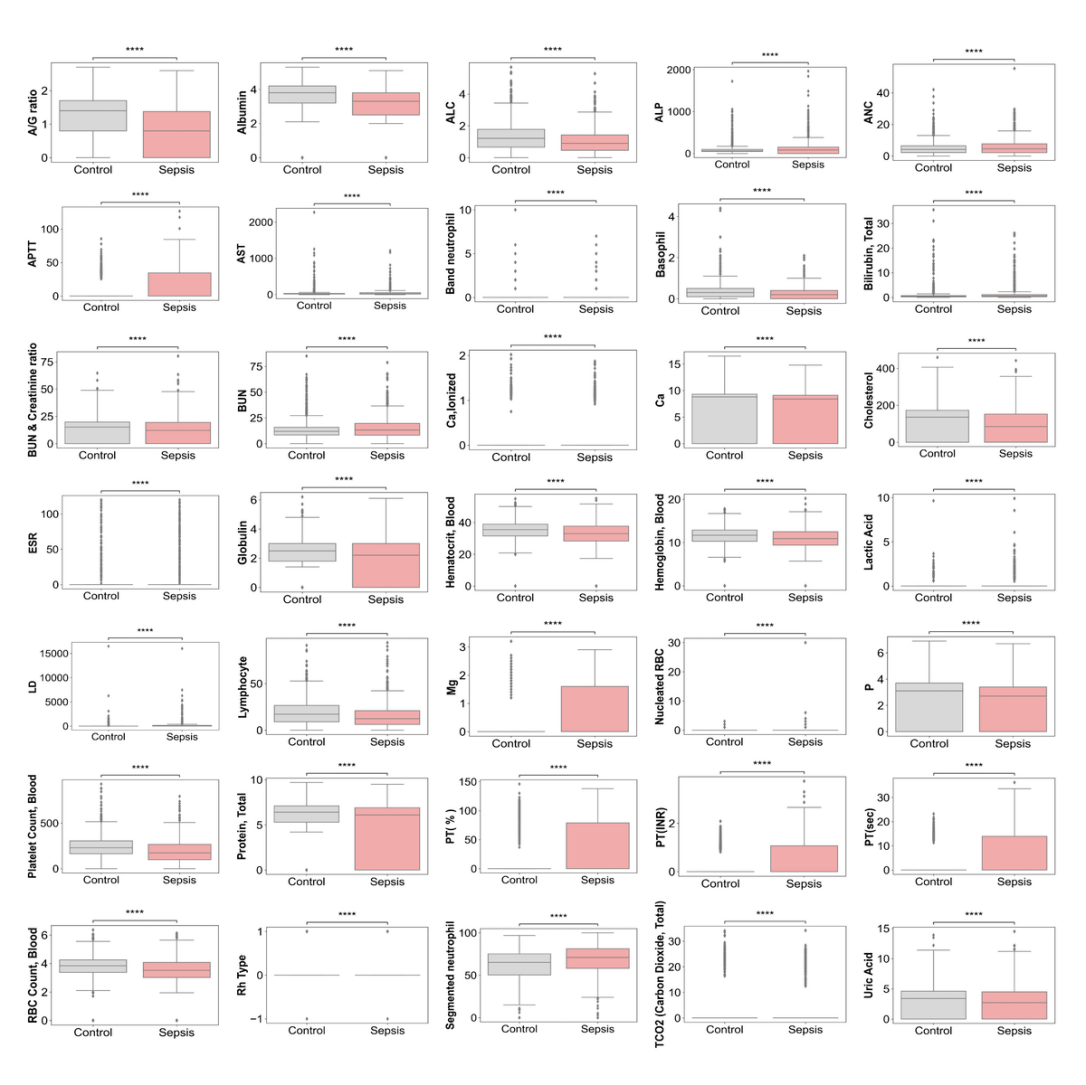
Distributions of the two groups for the selected lab test types. Of the 64 total lab test types (Multimedia Appendix 1), 35 lab test types showed significantly different distributions between the sepsis and control groups. ****: *P*<.001; A/G ratio: albumin/globulin ratio; ALC: absolute lymphocyte count; ALP: alkaline phosphatase; ANC: absolute neutrophil count; APTT: activated partial thromboplastin time; AST: aspartate aminotransferase; BUN: blood urea nitrogen; ESR: erythrocyte sedimentation rate; LD: lactate dehydrogenase; PT: prothrombin time; RBC: red blood cell.

Figure 3 presents the distributions of the 2 groups for our selected lab test types, including predictors that are well-known hallmarks of sepsis. The changes in albumin, total protein, and cholesterol levels reflected the higher risk of mortality in patients with sepsis [28], and in our results, the values of these factors were significantly lower in patients with sepsis, with an albumin level of 2.73 (SD 1.57) versus 3.3 (SD 1.47), total protein of 4.77 (SD 2.98) versus 5.27 (SD 2.82), and a cholesterol level of 81.06 (SD 84.79) versus 111.46 (SD 84.3), respectively, with *P*<.001. A decreased albumin/globulin ratio (A/G ratio) was recently reported as a novel independent predictor of the development of postflexible ureteroscopic sepsis [29]. In this study, the A/G ratio in the sepsis group was lower than that in the nonsepsis group, at 0.76 (SD 0.7) versus 1.14 (SD 0.69), respectively, with *P*<.001. Furthermore, several studies found that activated partial thromboplastin time (APTT) and prothrombin time (PT) [30,31] are prognostic biomarkers for the specific identification of patients with sepsis. We observed that the occurrence of sepsis led to increased APTT and PT values, with an *APTT* value of 6.89 (SD 15.01) to 14.06 (SD 19.72), *PT(%)* value of 17.51 (SD 35.82) to 33.27 (SD 42.26), *PT(INR)* value of 0.23 (SD 0.46) to 0.49 (SD 0.63), and *PT(sec)* value of 2.9 (SD 5.84) to 6.14 (SD 7.69), with *P*<.001. With favorable consistency between the identified lab test differences of our candidates and known biomarker candidates of sepsis, we felt confident that the SMC EMRs successfully recapitulated the physical and biological signatures of the patients with sepsis. In other words, these results suggested that the 35 selected biomarkers could characteristically reflect the biological features of the patients with sepsis. Thus, we utilized these 35 lab test results as learning features to establish our prediction model for sepsis, as shown in Figure 3.

### Prediction of Sepsis Using Machine Learning Approaches

Using vectorized drug relationships and the values of the selected lab test types along with common EHRs, we trained 2 machine learning models (logistic regression and random forest) and 3 deep learning models (ANN, convolutional neural network [CNN], and RNN) to build a sepsis prediction model based on the physical status of patients with cancer. A total of 465 relationships between 31 drugs selected through association rule analysis were vectorized using the 3 formulas described in the Methods section. A total of 1395 (465 × 3) drug relationship vectors, the values of the selected 35 lab test types, and common EHR information including anonymized personal information, hospitalization data, and cancer diagnosis code were used as inputs for model training. We used simple logistic regression (LR) and RNN-LSTM for the logistic regression–based and RNN-LSTM–based models, respectively. The random forest–based model comprised 20 trees, and the ANN-based model comprised input and output layers, as well as hidden layers. In addition, we used ResNet10 consisting of 10 convolution layers, fully connected layers, and residual connections for the CNN-based model. All hyperparameters, such as the number of trees in the random forest model, batch size, learning rate, and number of layers in the deep learning models, were selected as optimal values for each model through grid searches [32]. The list of feature variables used in our proposed model is given in Multimedia Appendix 3. To verify the proposed sepsis prediction model, we compared the predictive performances with the models trained on only common EHRs (ie, demography, diagnoses codes, and others) and the models trained on common EHRs and drug relationships by 5-fold cross-validation. Regarding performance evaluation metrics, the accuracy, area under the receiver operating characteristic (AUROC), area under the precision-recall curve (AUPRC), precision, recall, and F1 score were used. Multimedia Appendix 4 shows the performance evaluation results.

The overall performance of the proposed models with EHRs, lab data, and drug relationships were superior to that of the other models. The proposed random forest–based model showed the highest value in all the evaluation metrics except for recall (accuracy: 0.692, AUROC: 0.753, AUPRC: 0.573, precision: 0.518, recall: 0.718, and F1 score: 0.602). In the case of recall, the proposed ANN-based model showed the highest value (accuracy: 0.654, AUROC: 0.723, AUPRC: 0.522, precision: 0.477, recall: 0.721, and F1 score: 0.574). In particular, the proposed random forest–based proposed model recorded the largest performance improvement in all the metrics compared to the model trained on drug relationships and common EHRs (accuracy: 0.645 to 0.692, AUROC: 0.69 to 0.753, AUPRC: 0.487 to 0.573, precision: 0.465 to 0.518, recall: 0.629 to 0.718, and F1 score: 0.534 to 0.602). In addition, the proposed RNN-LSTM–based model showed the greatest performance improvement in accuracy, the AUROC, the AUPRC, and precision (accuracy: 0.603 to 0.675, AUROC: 0.655 to 0.729, AUPRC: 0.447 to 0.555, and precision: 0.43 to 0.504), and the ResNet10-based model showed the highest improvement in recall and the F1 score (recall: 0.577 to 0.689, F1 score: 0.499 to 0.567) compared to the model trained on only common EHRs. These findings suggest that the drug relationships and the selected lab test types were the main contributors to the proposed sepsis predictive models for patients with cancer.

### Investigation of Important Features

To evaluate the contributions of the learning features, SHAP, an XAI technique, was utilized for the proposed random forest–based model, which showed the best performance when investigating important features that contributed to the prediction. Multimedia Appendix 5 shows the contribution ratios of the top 50 important features among 1738 features obtained through SHAP, where the x-axis denotes the feature contribution ratio, and the y-axis denotes the names of the features.

The top 50 important features include 26 lab test types and 15 drug relationships among the 31 drugs and the 35 lab test types selected by *t* test and association rule analysis, respectively. The 15 drug relationships contained narcotic analgesic drugs such as “*opioid alkaloids*” and *“synthetic narcotics*,” which were prescribed more, along with other drugs, in the sepsis group. Among the characteristics of the patients with cancer, the number of cancer-infiltrating lymph nodes (Ca_LN_no), the degree of cancer extent (Extend_CD), and the size of the primary tumor (T_CD) were observed as decisive contributing factors.

As expected, prognostic biomarkers of sepsis, such as the albumin level, PT, A/G ratio, total protein level, and cholesterol level, ranked high. The blood platelet count has also been identified as a major contributor, and platelets are involved in mechanisms that promote immune responses and coagulation activation. Thrombocytopenia is common in ICU patients with sepsis and is reportedly associated with fatal outcomes [33]. The migration of neutrophils to infection sites is essential in the host’s defense against invading pathogens during sepsis [34], which may have led to the absolute neutrophil count or segmented neutrophils improving the predictive performance of the model. Moreover, when expanded to the top 100, all selected lab test types except *“band neutrophil*,” “*nucleated RBC,*” and “*carbon dioxide, total*,” as well as 49 drug relationships comprising 22 selected drugs, were included. These results show that the selected drug relationships and lab tests were important features in the proposed sepsis predictive model, suggesting that these features contributed to the accurate prediction of the model.

## Discussion

### Principal Findings

This study presents a machine learning–based approach to identify sepsis risk in patients with cancer at an early stage (2 days before onset). We elucidated that the relationships of prescribed medications and lab test patterns were distinct in the sepsis and control groups. Based on these analysis results, we built a machine learning–based sepsis prediction model trained on lab test items and vectorized drug relationships, along with EHRs. The proposed model outperformed the model trained on medication relationships or common EHRs. In particular, the proposed random forest–based model showed the best sepsis prediction performance (accuracy: 0.692, AUROC: 0.753, and F1 score: 0.602) and showed the greatest performance improvement. Furthermore, we demonstrated that the selected lab test results and drug relationships were indeed important features and mainly contributed to the accurate prediction of our proposed model. Therefore, lab tests and medication relationships can be used as efficient features for predicting sepsis. Consequently, it will be possible to use EHR information and deep learning methods to identify the risk of sepsis in patients with cancer.

### Limitations

Several limitations of the study should be noted. First, health records are not intended specifically for research; nonbilling-related data, including self-reported data such as smoking status, would be partially inaccurate. As depicted in Table 1, a substantial portion of patients with cancer are diagnosed with liver or lung cancer. Although there is a fairly significant incidence of liver and lung cancer in Korea [35], characteristic signatures of lab results and medications (eg, a lower A/G ratio and usage of opioid alkaloids) among patients with sepsis should be addressed at the pan-cancer level in further studies. For the contribution of the relationships of medication pairs, we acknowledge that there are many stakeholders in the prescription of medications, including insurance coverage. In this study, patients in Korea were all covered by the National Health Insurance Service. Thus, there would be a limited utilization of the relationship of medication combinations for model training in further applications from different countries corresponding to the heterogeneous milieu of insurance coverages.

As we hypothesized, our network-based analysis disclosed distinct patterns of medications used between sepsis and nonsepsis patients with cancer. For example, synthetic narcotics and opioid agents appeared to be more frequently prescribed with other agents. These features (ie, lab test results and medication patterns) mainly contributed to the high performance of our prediction model. Because the usage of opioids is a known risk factor for sepsis [36], the possibility of iatrogenic effects for the medication pattern–based prediction of sepsis in patients with cancer remains unclear. Therefore, drug-drug interactions between synthetic narcotics and anticancer agents should be addressed to further understand sepsis in patients with cancer. The retrospective analysis of EHRs paves the way for future research to understand sepsis among patients with cancer.

### Conclusion

To our knowledge, previous prognostic evaluation tools and models primarily use patient information obtained after admission to the ICU, and there are many limitations for medical interventions. However, since most patients with cancer are hospitalized through the emergency room for the initial diagnosis of sepsis, an appropriate evaluation tool is needed to identify the risk in advance. This study can be referenced as a baseline for efficiently predicting the onset of sepsis in patients with cancer, and the model is expected to be able to identify sepsis risk more accurately and earlier than before in the medical field.

## Appendix

Multimedia Appendix 1 Topological properties of the network.

Multimedia Appendix 2 Comparison of lab test numerical distribution in the sepsis versus control groups.

Multimedia Appendix 3 Description of the feature variables used in the proposed sepsis prediction model.

Multimedia Appendix 4 Performance evaluation results. (A-F) Comparison of the sepsis prediction performance for the models trained on only common EHRs (only EHRs), the models trained on common EHRs and drug relationships (EHRs+Drug Rel), and the models trained on drug relationships and lab test types, along with common EHRs (EHRs+Drug Rel+Lab test types (proposed)) obtained by 5-fold cross-validation. AUROC: area under the receiver operating characteristic; AUPRC: area under the precision-recall curve; EHR: electronic health record; LR: logistic regression; RF: random forest; ANN: artificial neural network; ResNet10: residual convolutional neural network with 10 layers; LSTM: long short-term memory recurrent neural network.

Multimedia Appendix 5 Feature contribution ratio of the Shapley Additive Explanations. Bar chart shows the feature contribution ratio (%) of the top 50 features obtained by the Shapley Additive Explanations algorithm for the random forest-based sepsis prediction model. A/G ratio: albumin/globulin ratio; ALP: alkaline phosphatase; AM: taking medications in the morning (ante meridiem); APTT: activated partial thromboplastin time; AST: aspartate aminotransferase; BUN: blood urea nitrogen; Ca_LN_no: number of cancer-infiltrating lymph nodes; Extend_CD: degree of cancer extent; IV: intravenous administration; T_CD: size of primary tumor; PT: prothrombin time; RBC: red blood cell.

## Abbreviations

A/G: albumin/globulin
ANN: artificial neural network
APTT: activated partial thromboplastin time
AUPRC: area under the precision-recall curve
AUROC: area under the receiver operating characteristic
CDW: Clinical Data Warehouse
CNN: convolutional neural network
EHR: electronic health record
ER: emergency room
FP-growth: frequent pattern growth
ICU: intensive care unit
KISTI: Korea Institute of Science and Technology Information
LR: logistic regression
NRF: National Research Foundation
PT: prothrombin time
ResNet10: residual convolutional neural networks
RF: random forest
RNN-LSTM: long short-term memory recurrent neural networks
Sepsis-3: Third International Consensus Definitions for Sepsis and Septic Shock
SHAP: Shapley Additive Explanations
SMC: Samsung Medical Center
SOFA: Sequential Organ Failure Assessment
XAI: Explainable Artificial Intelligence

## Data Availability

The data sets used and analyzed in this study are available from the corresponding author on reasonable request.
