# Supplementary figures and images for: Identifying the Risk of Sepsis in Patients With Cancer Using Digital Health Care Records: Machine Learning–Based Approach
Source: JMIR Med Inform. 2022 Jun 15;10(6):e37689. doi: 10.2196/37689 (PMC9244654; doi:10.2196/37689)

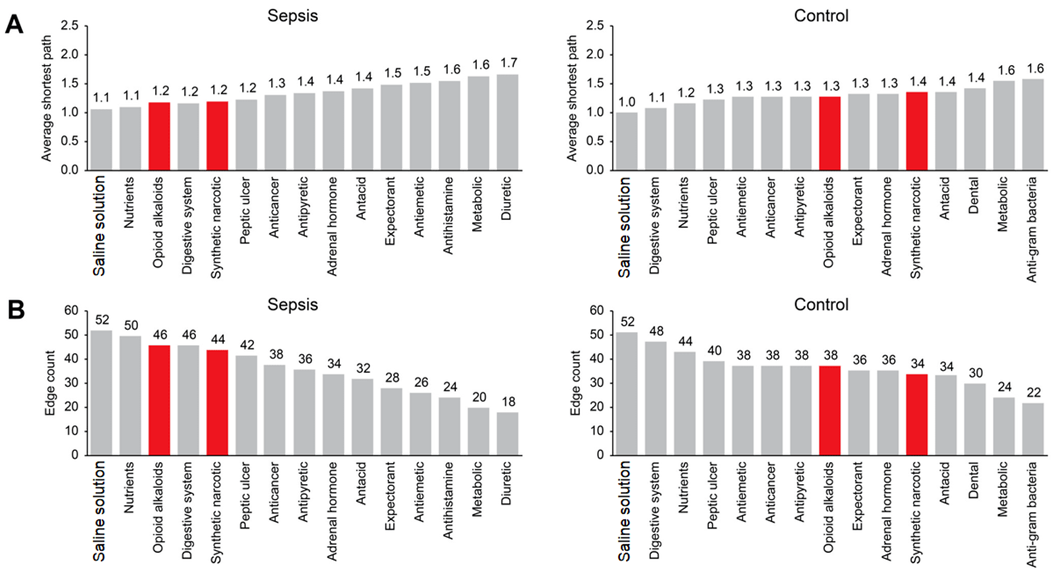

Supplement: Multimedia Appendix 1 [file medinform_v10i6e37689_app1.png]

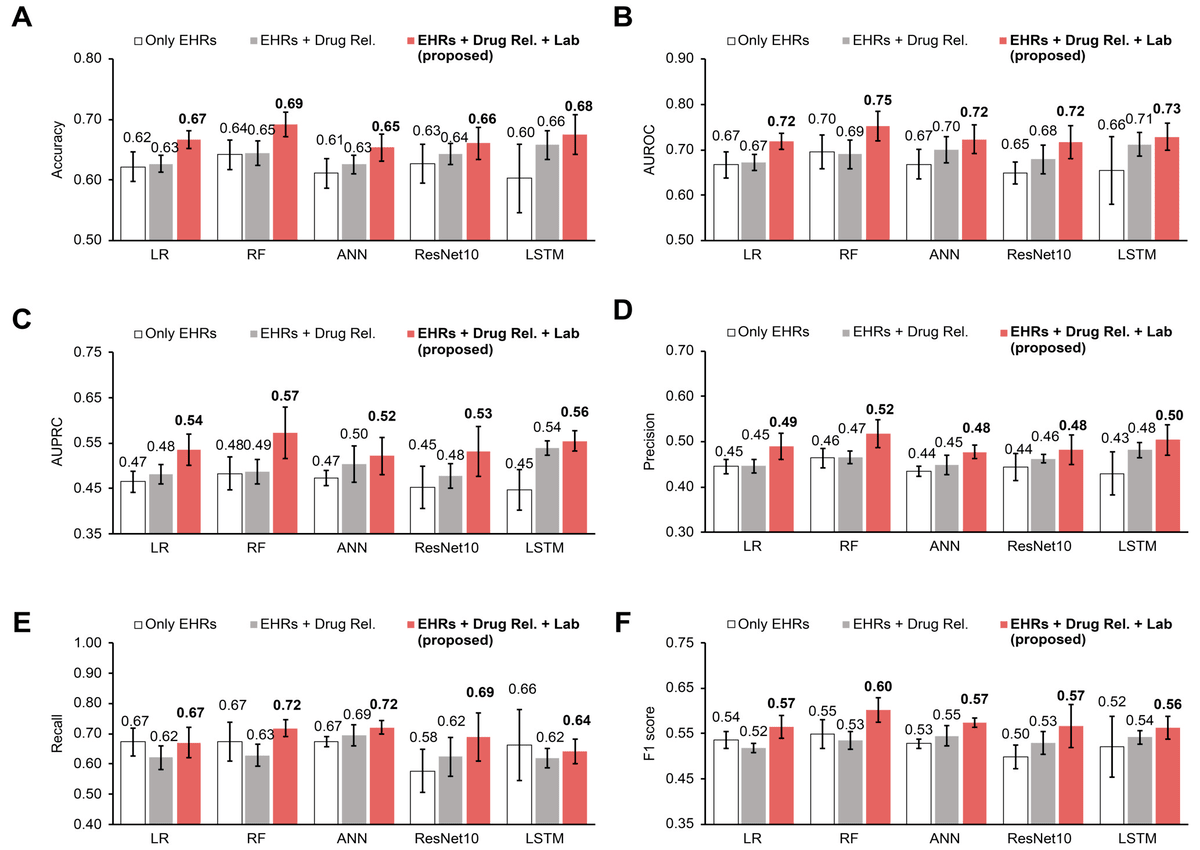

Supplement: Multimedia Appendix 4 [file medinform_v10i6e37689_app4.png]

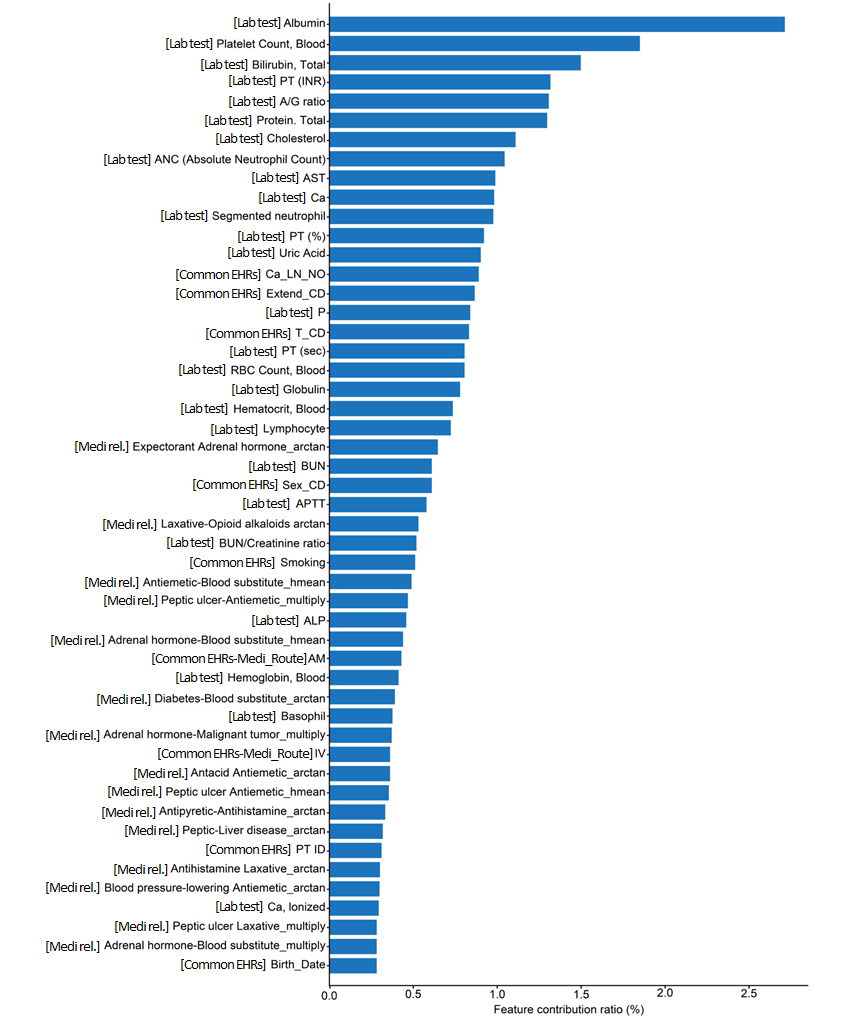

Supplement: Multimedia Appendix 5 [file medinform_v10i6e37689_app5.png]
