# Supplementary material for: Identifying the Risk of Sepsis in Patients With Cancer Using Digital Health Care Records: Machine Learning–Based Approach
Source: JMIR Med Inform. 2022 Jun 15;10(6):e37689. doi: 10.2196/37689 (PMC9244654; doi:10.2196/37689)
Supplement: Multimedia Appendix 2 [file medinform_v10i6e37689_app2.docx]

**Multimedia Appendix 2. Comparison of lab test numerical distribution in the sepsis vs. control groups**

| Lab test types | Control group (mean ± std) | Sepsis group (mean ± std) | *P* |
| --- | --- | --- | --- |
| A/G ratio | 1.14 ± 0.69 | 0.76 ± 0.7 | **** |
| Abnormal lymphoid cell | 0.0 ± 0.0 | 0.0 ± 0.0 | N.S. |
| ABO group RBC | 0.0 ± 0.0 | 0.0 ± 0.0 | N.S. |
| Albumin | 3.3 ± 1.47 | 2.73 ± 1.57 | **** |
| ALC | 1.25 ± 0.86 | 1.0 ± 0.78 | **** |
| ALP | 87.75 ± 108.76 | 129.02 ± 183.23 | **** |
| ALT | 35.95 ± 66.79 | 42.34 ± 85.34 | * |
| ANC | 4.77 ± 4.06 | 5.54 ± 5.06 | **** |
| Antibody screen | 0.0 ± 0.0 | 0.0 ± 0.0 | N.S. |
| Anti-HBs antibody | 7.12 ± 72.98 | 8.32 ± 83.34 | N.S. |
| Anti-HCV antibody | -0.03 ± 0.44 | -0.01 ± 0.5 | N.S. |
| Anti-HIV combo | -0.04 ± 0.21 | -0.03 ± 0.18 | N.S. |
| APTT | 6.89 ± 15.01 | 14.06 ± 19.72 | **** |
| AST | 38.78 ± 88.01 | 54.55 ± 98.23 | **** |
| Atypical lymphocyte | 0.01 ± 0.11 | 0.02 ± 0.19 | ** |
| Band neutrophil | 0.05 ± 0.39 | 0.15 ± 0.63 | **** |
| Basophil | 0.35 ± 0.36 | 0.3 ± 0.34 | **** |
| Bilirubin, total | 0.68 ± 1.82 | 1.33 ± 2.78 | **** |
| Blast | 0.0 ± 0.0 | 0.0 ± 0.04 | * |
| BUN | 12.43 ± 8.68 | 14.45 ± 11.13 | **** |
| BUN/creatinine ratio | 13.65 ± 9.93 | 11.43 ± 11.93 | **** |
| Ca | 6.62 ± 4.07 | 5.76 ± 4.34 | **** |
| Ca, ionized | 0.06 ± 0.28 | 0.24 ± 0.49 | **** |
| Cholesterol | 111.46 ± 84.3 | 81.06 ± 84.79 | **** |
| Cl | 66.72 ± 47.26 | 72.1 ± 44.18 | ** |
| Creatinine | 0.71 ± 0.68 | 0.73 ± 0.56 | N.S. |
| CRP quantitative | 1.97 ± 3.97 | 2.26 ± 4.94 | N.S. |
| Eosinophil | 1.74 ± 2.33 | 1.55 ± 2.45 | * |
| ESR | 2.52 ± 13.61 | 9.61 ± 26.42 | **** |
| Estimated GFR | 79.53 ± 39.65 | 77.09 ± 47.62 | N.S. |
| Globulin | 2.14 ± 1.3 | 1.78 ± 1.6 | **** |
| Glucose, fasting | 94.08 ± 66.17 | 90.31 ± 74.32 | N.S. |
| HBsAg | 36.17 ± 442.97 | 31.41 ± 395.59 | N.S. |
| Hematocrit, blood | 33.72 ± 9.84 | 31.57 ± 10.28 | **** |
| Hemoglobin, blood | 11.13 ± 3.3 | 10.44 ± 3.44 | **** |
| Immature cell | 0.0 ± 0.02 | 0.01 ± 0.09 | ** |
| Lactic acid | 0.03 ± 0.3 | 0.25 ± 0.79 | **** |
| LD | 82.56 ± 410.03 | 224.26 ± 773.52 | **** |
| Lymphocyte | 18.31 ± 13.16 | 14.97 ± 12.88 | **** |
| MCH | 28.79 ± 7.52 | 28.77 ± 8.04 | N.S. |
| MCHC | 31.04 ± 7.87 | 30.87 ± 8.34 | N.S. |
| MCV | 87.24 ± 22.52 | 87.02 ± 24.06 | N.S. |
| Metamyelocyte | 0.04 ± 0.36 | 0.08 ± 0.41 | ** |
| Mg | 0.24 ± 0.66 | 0.55 ± 0.9 | **** |
| Monocyte | 7.64 ± 5.1 | 7.29 ± 5.09 | N.S. |
| Myelocyte | 0.02 ± 0.21 | 0.02 ± 0.17 | N.S. |
| Na | 91.91 ± 65.02 | 98.98 ± 60.45 | ** |
| Nucleated RBC | 0.01 ± 0.11 | 0.09 ± 1.0 | **** |
| P | 2.49 ± 1.6 | 2.17 ± 1.59 | **** |
| Plasma cell | 0.0 ± 0.0 | 0.0 ± 0.0 | N.S. |
| Platelet count, blood | 236.48 ± 123.03 | 189.15 ± 127.04 | **** |
| Potassium | 2.88 ± 2.07 | 3.12 ± 1.96 | ** |
| Promyelocyte | 0.0 ± 0.0 | 0.0 ± 0.0 | N.S. |
| Protein, total | 5.27 ± 2.82 | 4.77 ± 2.98 | **** |
| PT (%) | 17.51 ± 35.82 | 33.27 ± 42.26 | **** |
| PT(INR) | 0.23 ± 0.46 | 0.49 ± 0.63 | **** |
| PT (sec) | 2.9 ± 5.84 | 6.14 ± 7.69 | **** |
| RBC count, blood | 3.65 ± 1.09 | 3.4 ± 1.13 | **** |
| Rh type | 0.09 ± 0.29 | 0.16 ± 0.37 | **** |
| RPR, quantitative | 0.0 ± 0.01 | 0.0 ± 0.01 | N.S. |
| Segmented neutrophil | 57.2 ± 27.04 | 63.14 ± 27.38 | **** |
| TCO_2_ | 1.18 ± 5.16 | 3.31 ± 7.89 | **** |
| Uric acid | 3.08 ± 2.31 | 2.69 ± 2.58 | **** |
| WBC count, blood | 7.56 ± 4.54 | 7.73 ± 5.58 | N.S. |

*P: P value; ****: P < 0.0001; ***: P < 0.001; **: P < 0.005; *: P < 0.05; N.S.: No Significance.*

ALC: absolute lymphocyte count; ALP: alkaline phosphatase; ALT: alanine aminotransferase; ANC: absolute neutrophil count; APTT: activated partial thromboplastin time; AST: aspartate aminotransferase; BUN: blood urea nitrogen; ESR: erythrocyte sedimentation rate; LD: lactate dehydrogenase; MHC: mean corpuscular hemoglobin; MCHC: mean corpuscular hemoglobin concentration; MCV: mean corpuscular volume; PT: prothrombin time; RBC: red blood cell; TCO_2_: Total carbon dioxide; WBC: white blood cell.
