# Supplementary material for: Identifying the Risk of Sepsis in Patients With Cancer Using Digital Health Care Records: Machine Learning–Based Approach
Source: JMIR Med Inform. 2022 Jun 15;10(6):e37689. doi: 10.2196/37689 (PMC9244654; doi:10.2196/37689)
Supplement: Multimedia Appendix 3 [file medinform_v10i6e37689_app3.docx]

**Multimedia Appendix 3. Description of the feature variables used in the proposed sepsis prediction model.**

| Category | Idx. | Variable | Description |
| --- | --- | --- | --- |
| Common  EHRs | 1 | PT_ID | Patient index |
|  | 2 | SMC_Dx_Date | Date of cancer diagnosis at SMC  (Period from initial cancer diagnosis to the cancer diagnosis at SMC) |
|  | 3 | Age | Patient age |
|  | 4 | Sex_CD | Patient gender (Male: 0, Female: 1) |
|  | 5 | Wt | Patient weight |
|  | 6 | Smoking | Smoking: 1, Non-smoking: 0 |
|  | 7 | Extend_CD | Degree of cancer extent (0~7) |
|  | 8 | Ca_LN_No | Number of cancer-infiltrating lymph nodes |
|  | 9 | T_CD | Size of primary tumor (0~4) |
|  | 10 | N_CD | Lymph node status (0~3) |
|  | 11 | M_CD | Distant metastasis: 1, No distant metastases: 0 |
|  | 12  ~  14 | Primary_organ | One-hot encoded primary site of cancer, referenced in ICD-O-3 code  (C22: Liver, C34: Lung, C50: Breast) |
|  | 15  ~  99 | Histo_Dx_CD | One-hot encoded histological diagnosis code referenced in ICD-O-3 code (M8000, M8004, M8010, M8012, M8013, M8020, M8022, M8031, M8032, M8033, M8041, M8045, M8046, M8052, M8070, M8071, M8072, M8073, M8075, M8082, M8083, M8123, M8140, M8141, M8160, M8162, M8170, M8180, M8200, M8201, M8211, M8230, M8240, M8246, M8249, M8253, M8255, M8260, M8265, M8401, M8430, M8480, M8481, M8490, M8500, M8501, M8502, M8503, M8507, M8510, M8520, M8522, M8523, M8541, M8550, M8560, M8562, M8570, M8574, M8575, M8800, M8801, M8802, M8805, M8811, M8825, M8890, M9020, M9040, M9050, M9052, M9120, M9133, M9364, M9590, M9663, M9680, M9684, M9687, M9699, M9702, M9714, M9724, M9751, M9930) |
|  | 100 | Histo_Dx_be | Histological behavior code of cancer  (in situ, Intraepithelial, Noninfiltrating, Noninvasive): 2, Malignant: 3 |
|  | 101  ~  199 | Medi_Days | One-hot encoded medication type * Number of prescribed days  (Sedative, Antipyretic, Psychoneurotic, Antihistamine, Diuretic, Blood ressure lowering, Blood circulatory, Expectorant, Dental, Peptic ulcer, Peptic, Antacid, Antiemetic, Laxative, Digestive system, Adrenal hormone, Vitamin B, Nutrients, Protein, Blood substitute, Anticoagulant, Body fluids, Liver disease, Diabetes, Metabolic, Malignant tumor, Anticancer, Anti-gram bacteria, Chemotherapy, Opioid alkaloids, Synthetic narcotic, Acid-fast bacterium, Anti-allergic, Antiarrhythmic, Antibiotic, Anticancer2, Anticonvulsants, Antidote, Antifungal, Anti-gram-negative bacteria, Anti-gram-negative bacteria2, Anti-gram-positive bacteria, Anti-inflammatory, Antiprotozoal, Antispasmodics, Antituberculosis, Arteriosclerosis, Arthrifuge, Autonomic, Blood product, Calcium, Cardiotonic, Central nervous system, Cholagogue, Contrast agent, Cutaneous, Drug from outside, Enzyme inhibitor, Follicle and luteal hormone, General Anesthetics, Hair, Hemorrhoid, Hemostatic , Hormone, Immunomodulators, Laxative, Local anesthetic, Mineral, Multi Vitamins, Nourishing tonic, Ointment, Ophthalmic, Others for non-treatment, Others for treatment, Otorhinolaryngological, Perfusate, Pituitary hormone, Probiotics, Psychostimulant, Reagents for functional, Reagents for general , Relaxant, Resolvent, Respiratory, Respiratory2, Skin, Skin softener, Sterilization and Disinfection, Thyroid and parathyroid hormone, Tissue cell, Urinary and anal, Vaccines, Vasodilator, Vasohypertonic, Vasohypotonic, Vitamin, Vitamin AD, Vitamin BI, Vitamin EK)  *Note that this variable includes the drugs (first 31) selected through association rule and used to generate drug relationship variables. |
|  | 200  ~  308 | Medi_Route | One-hot encoded route of medication administration  (A total of 109 routes including Intravenous (IV), By mouth (PO), Before noon (AM), #Hour (H), Every hour (QH), Before sleep (HS).) |
| Lab test | 1  ~  35 | Lab_test | One-hot encoded lab test selected by T-test in this study * Result value (A/G ratio, Albumin, ALC, ALP, ANC, APTT, AST, Band neutrophil, Basophil, Bilirubin Total, BUN, BUN&Creatinine ratio, Ca, Ca Ionized, Cholesterol, ESR, Globulin, Hematocrit Blood, Hemoglobin Blood, Lactic Acid, LD, Lymphocyte, Mg, Nucleated RBC, P, Platelet Count Blood, Protein Total, PT(%), PT(INR), PT(sec), RBC Count Blood, Rh Type, Segmented neutrophil, TCO2(Carbon Dioxide, Total), Uric Acid) |
| Medication relationship | 1  ~  465 | Medi_multiply_Medi | Vectorized relationships by the interaction (*I*) of Equation (2) between 31 drugs selected through association rule |
|  | 466  ~  930 | Medi_hmean_Medi | Vectorized relationships by the harmonized average (*H*) of Equation (2) between 31 drugs selected through association rule |
|  | 931  ~  1,395 | Medi_arctan_Medi | Vectorized relationships by the arctangent (*T*) of Equation (2) between 31 drugs selected through association rule |
